# The role of color contrast gain control in global form perception

**DOI:** 10.1167/jov.22.6.11

**Published:** 2022-05-31

**Authors:** Yih-Shiuan Lin, Lee Lin, Chien-Chung Chen

**Affiliations:** 1 Institute of Experimental Psychology, University of Regensburg, Regensburg, Germany; 2 Department of Psychology, National Taiwan University, Taipei, Taiwan; 3 Neurobiology and Cognitive Science Center, National Taiwan University, Taipei, Taiwan

**Keywords:** glass pattern, color contrast, contrast gain control, perceptual grouping, object recognition

## Abstract

A Glass pattern consists of randomly distributed dot pairs, or dipoles, whose orientation is determined by a geometric transform, which defines the global percept perceived by an observer. The perception of Glass patterns involves a local process that associates dot pairs into dipoles and a global process that groups the dipoles into a global structure. In the present study, we used a variant of Glass patterns, which was composed of randomly distributed tripoles instead of dipoles, to estimate the influence of color contrast on perceptual grouping. Each tripole contained an anchor dot and two context dots. Grouping the anchor dot with one of the context dots resulted in a global percept of a clockwise spiral, while grouping with the other dot, a counter-clockwise spiral. All dots in each pattern were modulated in the same color direction but different contrasts. Four colors were involved, namely, red, green, blue, and yellow. The observers were to determine whether the spiral in each trial was clockwise or counter-clockwise. The probability of a context dot being grouped with the anchoring dot increased with its color contrast to a certain level, then decreased when the contrast continued to increase. Such probability decreased as the contrast of the other context dot increased. Our result cannot be explained by existing models in the literature, but with a divisive inhibition model. The equiluminance contrast result observed here is similar to the inverted U-shaped function for luminance contrast result previously reported by us, except that the color contrast model comprises a weaker self-inhibition component.

## Introduction

The major function of vision is to recognize objects in a scene. Given that the input image has been decomposed into fragmented lines segmented by the early visual system, which contains orientation-selective mechanisms with localized receptive fields, such a global percept for objects is possible only if the visual system is able to integrate local image elements together to form the percept of coherent entities. Such integration, or perceptual grouping, processes have been well studied since the time of Gestalt Psychology (Köhler, 1970). Many grouping phenomena have been described, and many grouping “laws,” such as proximity, similarity, or closure, have been identified (Metzger, 2006).

Here, we are interested in the effect of chromatic information in perceptual grouping processes. In general, the role of color in form and object perception is still elusive. There is evidence suggesting that color plays a secondary, if any, role in analyzing spatial patterns. The contrast sensitivity functions measured with equiluminance stimuli showed a low-pass characteristics (Mullen, 1985), different from the band-pass contrast sensitivity function measured with luminance-modulated stimuli. Many color appearance phenomena, such as neon color illusion (Redies & Spillmann, 1981; van Tuijl, 1975), or watercolor illusion (Pinna, Brelstaff, & Spillmann, 2001; Pinna & Grossberg, 2005), occur because it is difficult for an observer to perceive the boundary of a colored stimulus. Li and Guo (1995) examined the strength of several visual illusions under achromatic and equiluminance contrast, and showed that the geometric illusions (e.g. the Zöllner illusion, Müller-Lyer illusion, and Ponzo illusion) were comparable in equiluminance and isochromatic conditions. However, in Kaniza patterns, the equiluminance inducers failed to induce the illusory contours, whereas the inducers with even a low luminance contrast were sufficient to produce an illusion (Li & Guo, 1995).

On the other hand, there is also plenty of evidence showing that the color vision mechanisms also have the ability for a precise spatial pattern analysis suggestion (for a review, see De Valois, 2004). It is well documented that the visual system shows spatial-frequency or orientation-tuning properties for equiluminance stimuli (Beaudot & Mullen, 2005; Clifford, Pearson, Forte, & Spehar, 2003; Webster, De Valois, & Switkes, 1990), suggesting an ability for analyzing spatial information in an image.

The role of color in perceptual grouping is rather controversial. For instance, in symmetry perception, an important perceptual grouping phenomenon, Pashler and colleagues (Huang & Pashler, 2002; Morales & Pashler, 1999) argued that symmetry perception mechanisms are color blind. On the other hand, with a noise-masking paradigm, Wu and Chen (Wu & Chen, 2014) showed that symmetry detection is color specific. Gheorghiu and colleagues suggested that different stages of symmetry processing may have different color selectivity properties (Gheorghiu, Kingdom, Remkes, Li, & Rainville, 2016). In contour integration, an observer can integrate equiluminance Gabor elements into a contour (McIlhagga & Mullen, 1996; Mullen, Beaudot, & McIlhagga, 2000). However, the integration performance declined when local elements alternated between isochromatic and equiluminance Gabor patches, suggesting separate mechanisms for luminance and color, respectively, in contour integration.

Mullen and Beaudot used chromatic and achromatic radial frequency patterns (H. R. Wilson & Wilkinson, 1997) as stimuli in a 2AFC shape discrimination task, in which participants were to discriminate between circular and non-circular radial frequency patterns (Mullen & Beaudot, 2002). They discovered that the radial modulation threshold was the highest in blue-yellow, followed by red-green, and the lowest in achromatic condition. Their results demonstrate that color alone can support global shape processing of the radial frequency patterns, although with poorer performances compared to luminance, suggesting that the shape analysis mechanism might be different for color and luminance systems.

Glass patterns are composed of randomly distributed dot pairs (dipoles), whose orientation conforms to certain geometric transforms to give a percept of a global form. To perceive the global form, the visual system should contain at least two stages of operation: the first is to assess the orientation of local dipoles, and the second is to integrate local dipoles for the global form (Cardinal & Kiper, 2003; Chen, 2009; Dakin & Bex, 2001; Li & Chen, 2011; Mandelli & Kiper, 2005; Wilson, Wilkinson, & Asaad, 1997; Wilson, Switkes, & De Valois, 2004). Kiper and colleagues (Cardinal & Kiper, 2003; Mandelli & Kiper, 2005) manipulated dot colors in Glass patterns and measured the coherent threshold, or, given the same total number of dipoles in the image, the minimal number of dipoles that is needed for participants to perceive the global form among randomly-orientated dipoles. They estimated the color tuning in the local stage by varying the color difference between two dots in each dipole. As the color difference increased, the performance deteriorated, suggesting a color-selective local stage. For the integration stage, they varied the color difference between signal and noise dipoles and found that the coherence threshold was higher when the noise was of the same color as the pattern, and decreased as the color difference between the signal and noise increased. This was true not only for signal colors in the cardinal directions, but intermediate directions. Their results suggested that both stages of Glass pattern perception are color-selective, with the first more selective than the second.

**Figure 1.**
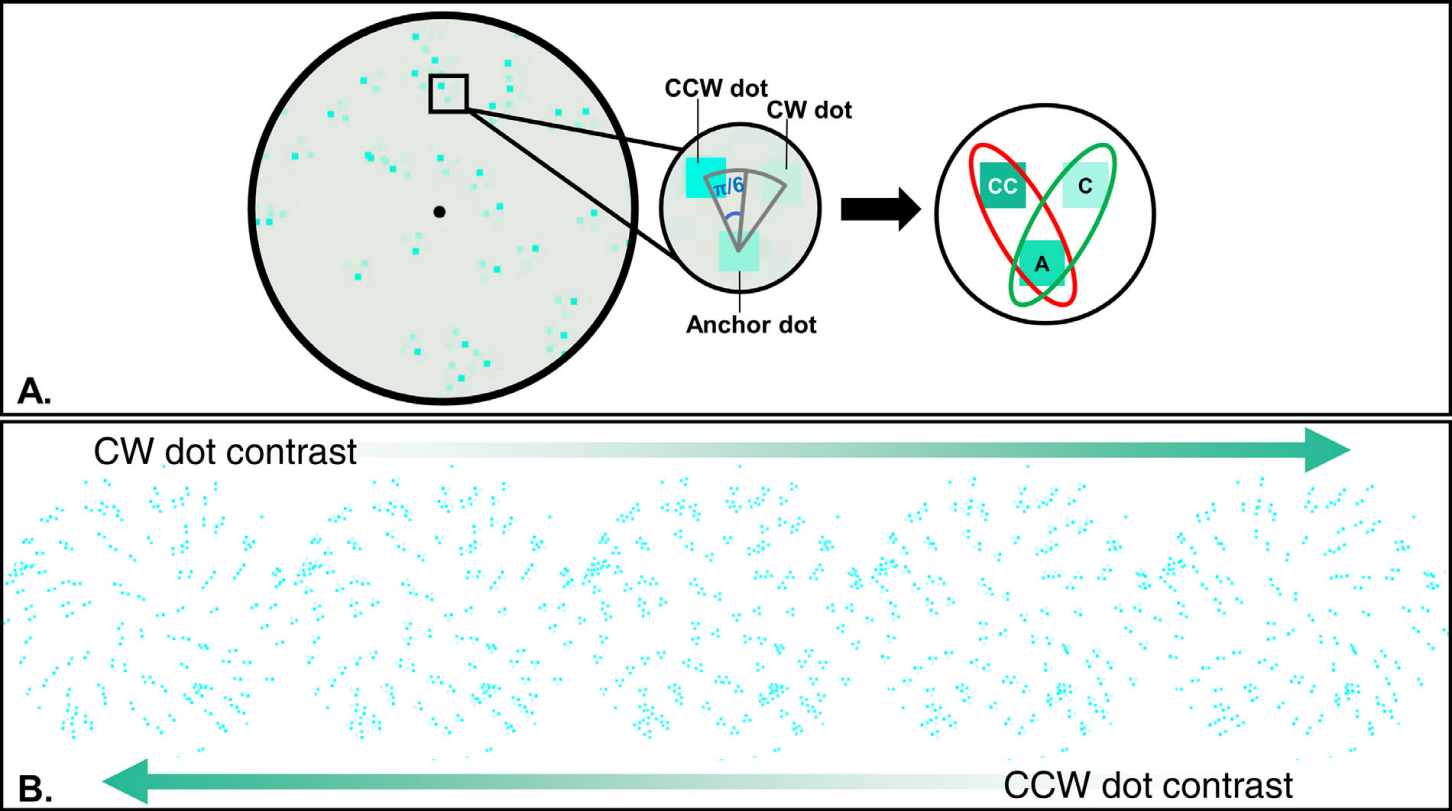
Illustration of one tripole Glass Pattern. Note: This figure shows examples of tripole Glass Pattern (tGP). Panel (**A**) represents a zoomed in version of one tGP. This version of the Glass pattern is composed of multiple local “tripoles.” Locally, a tripole (the zooming-in image in the middle circle) comprises three equal-size dots positioned in equal distance. The anchor dot (“**A**”) can be grouped with a counter-clockwise (CCW) dot (“**CC**”) that results in a CCW dipole (red oval). All CCW dipoles in the tGP lead to a global CCW spiral percept. When the anchor dot instead is grouped with the clockwise (CW) dot (“**C**”), a CW dipole is formed. All CW dipoles in the tGP lead to a global CW spiral percept. Panel (**B**) demonstrates how the tGP global percept changes with the CW and CCW dot contrasts. From left to right, the CW dot contrast increases while the CCW dot contrast decreases, resulting in a reversed percept from a CCW spiral to a CW spiral.

Here, we are interested in quantitatively characterizing the response properties of the color-selective mechanisms in the perceptual grouping process. We adapted the tripole Glass patterns (tGPs) paradigm (Lin, Cho, & Chen, 2017) for this purpose. Unlike conventional Glass patterns (Glass, 1969; Glass & Pérez, 1973), which consist of random dot pairs (or dipoles), a triple Glass pattern (Figure 1) was made of sets of three dots, or tripoles. Each tripole consisted of three dots, one anchor dot, as well as two context dots. Grouping the anchor dot with one context dot would produce a global percept of a counter-clockwise (CCW) spiral, whereas grouping the anchor dot with the other would produce a percept of a clockwise (CW) spiral. For the convenience of discussion, the former is called a CCW dot, whereas the latter is called a CW dot. Lin et al. (2017) asked observers to judge whether a tGP was a CCW or CW spiral. Thus, although it is possible for an observer to group the two context dots together, this grouping would not yield a relevant global form percept for the task.

Lin et al. (2017) found that the probability of the CW spiral percept first increased, then decreased, with the CW dot contrast, and that the CW spiral judging probability decreased with the contrast of the CCW dot. This suggested a competition between the two global groupings. Neither the energy model, which would predict that the anchor dot was grouped with whichever dot that had the greatest contrast (Prazdny, 1984), nor the similarity or token model, which would predict that the anchor dot was grouped with the dot of the same contrast level (Earle, 1999; Wilson et al., 2004), can fully explain the results. Instead, Lin and colleagues concluded that the grouping performance is subject to a contrast gain control process that takes the luminance of all dots into account. They proposed a version of the divisive inhibition model to explain their result (Lin et al., 2017).

Here, we extended the Lin et al. (2017) paradigm to test whether the color mechanisms for perceptual grouping have similar gain control properties. Instead of manipulating the dot luminance contrast in tGPs, we changed the dot color contrast while keeping their luminance at a constant. By doing so, we could assess how the global form percept changes with the local chromaticity variation. The visual mechanisms for luminance and color information showed different spatial properties as reviewed before. By comparing the results from that of Lin et al. (2017) and the current study, we can thus examine the gain control characteristic differences for grouping by luminance and chromaticity.

## Methods

### Participants

Four observers (2 women) participated in this experiment, including two of the authors (YSL and LL) and two other participants (NP1 and NP2), who were naïve to the purpose of this experiment. All the observers had normal (20/20) or corrected-to-normal visual acuity. Author YSL and NP1 completed all chromatic conditions (red, green, blue, and yellow), whereas NP2 and author LL completed parts of the conditions (NP2: all but the low-contrast anchor red/green conditions; LL: only low-contrast anchor red and blue conditions). YSL and LL also took part in the threshold measurement and subjective equiluminance control experiment (see Appendix). The study was approved by the Research Ethics Committee of National Taiwan University (approval number: NTU-REC 201405HM039). Written consent was obtained from each observer before the experiment.

### Apparatus

A 24-inch EIZO LCD monitor (FlexScan SX2462W) with a resolution of 1920 × 1200 was used for stimuli presentation. A Macintosh computer through an ATI Radeon HD 4870 graphics board, providing 10-bit, digital-to-analog converter depth, controlled the monitor. The refresh rate of the LCD monitor was 60 Hz. The luminance and chromaticity of the monitor were calibrated with a PhotoResearch PR655 radiometer. The display showed a mean luminance of 69.6125 cd/m^2^ and mean chromaticity at (0.3354 and 0.3342) in CIE 1931-xy coordinates. The viewing distance between observer and the center of the monitor was set such that each pixel extended 1 minute of visual angle.

### Stimuli

A tGP composed of three-dot sets, or “tripoles” (see Figure 1), was used in this study. To generate a tGP, we first drew randomly distribute square dots (7 inch × 7 inch visual angle) in the stimulus field as anchor dots (illustrated as the square denoted “A”). We chose relatively larger dots than those used in previous isochromatic experiments (Chen, 2009; Li & Chen, 2011) to accommodate the lower spatial-frequency tuning of color-vision mechanisms, and to avoid longitude chromatic aberration. Context dots (CCW and CW dots) that had the same size as the anchor dot were placed on either side of the radial line passing through the anchor dot. The line that passed through a context dot and the anchor dot intercepted the radial line with an angle π/6. Thus, three dots in a tripole formed the vortices of an equilateral triangle, with the anchor dot pointing toward the center of the display. The distance between two dot centers in a tripole was 17 inches. The dot density, or the proportion of area occupied by the dots, was 4%. Such relatively low density should reduce the overlapping between tripoles. The overall size of a tGP was 10 degrees visual angle.

The chromaticity was defined in a cone contrast space (Brainard, 1996). Here, we chose four colors, labeled red, green, blue, and yellow. We understand that these terms were not the most precise description of these colors. However, they are convenient for the communication with the participants. The chromaticity of each dot was defined by a contrast vector with three contrast values, *C =* *(C_L_, C_M_, C_S_)*, *C_L_* being the L-cone contrast, *C_M_* the M-cone contrast, and *C_S_* the S-cone contrast. The L-cone contrast, *C_L_*, was defined as *ΔL/L_0_*, where *L_0_* representing the L-cone excitation produced by the background, and *ΔL* the increments or decrements of the L-cone excitation produced by the dot. The M-cone and S-cone contrasts, *C_M_* and *C_S_*, were defined similarly. Cone excitations were estimated as the product of the spectral power distribution of the input light and the spectral sensitivity functions of each cone (Stockman & Sharpe, 2000). Eventually, each contrast vector was composed of a scalar value for contrast and a normalized cone contrast vector, *C / ||C||*, where *||C||* denotes the length of the vector *C*. In the present study, the normalized cone contrast vectors used were (0.416, −0.909, and 0) for red, (−0.416, 0.909, and 0) for green, (0, 0, and 1) for blue, and (0, 0, and −1) for yellow. Note that the normalized cone contrast vectors for equiluminance stimuli were all orthogonal to the CIE 2006 luminous efficiency function *V_λ_* (CIE, 2006), which corresponded to the normalized vector (0.853, 0.522, and 0). The final contrast of each dot was defined as *C = (C_L_^2^ + C_M_^2^ + C_S_^2^)^0.5^/3^0.5^*, proportional to the square root of the cone contrast energy and varied between 0 and 1. Contrast was expressed in dB, which equaled 20*log_10_C, ranged from −∞ to 0.

For each tGP presented, all dots had either one of the four hues. Because human L and M cones have higher input sensitivity than S cones, and easily become saturated under high contrast, we chose contrast levels based on the detection thresholds of these colors in a way that contrasts covered from near threshold level to the maximum contrast that can be produced by our apparatus. The detail of threshold measurement can be found in Appendix A. Thus, for red and green, contrasts of context dots ranged from −35 dB to −12 dB, whereas anchor dot contrast was either −30 dB or −18 dB. For blue and yellow, context dots contrasts ranged between −13 dB and −1 dB. The anchor dots were always kept at −7 dB. The luminance of all dots matched that of the background (mean luminance), so that luminance contrast would not contaminate the effect of chromatic contrast.

### Procedure

We used a 2AFC (two-alternative forced-choice) paradigm. In each trial, after an auditory cue indicating the start of a new trial, one tGP was presented on the monitor for a duration of 500 ms, together with a fixation point in the center. Participants were to decide whether the pattern was a CW or a CCW spiral by pressing the corresponding key. The next trial would not start until 800 ms after the response was recorded. The trials were organized into runs. In each run, all combinations of context dot contrasts for a particular anchor chromaticity were presented four times in a random sequence. There were 10 runs for each anchor. Thus, overall, there were 40 measurements for each test condition. We then sorted the data and reported the probability of pressing the CW key under each contrast combination.

## Results

### Red and green colors

**Figure 2.**
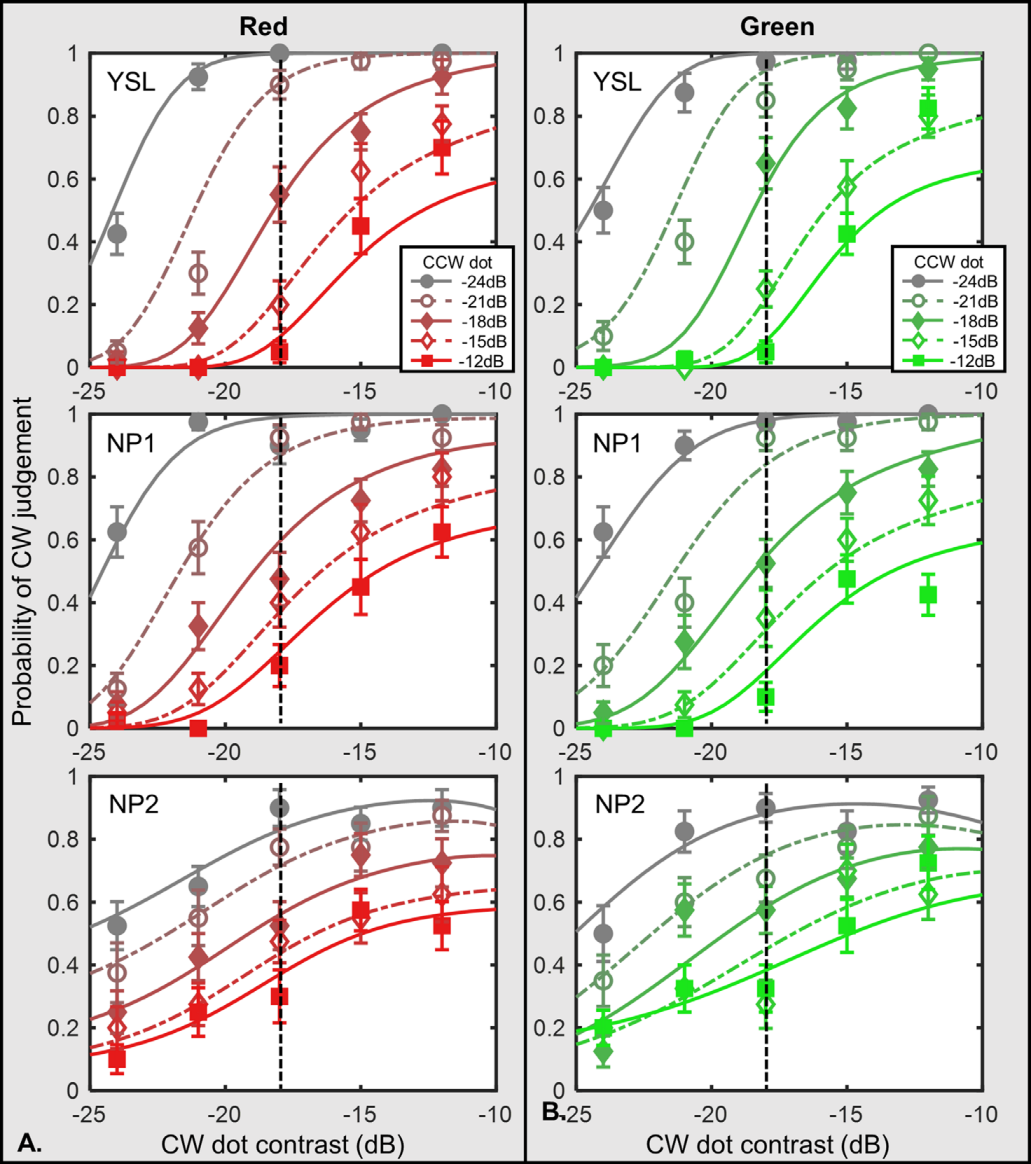
Results of red/green conditions with high-contrast anchor dots. Note: This figure shows the results of the red and green conditions with a −18 dB anchor dot. (**A**) Data from three observers of the red condition. (**B**) Data from three observers of the green condition. Probability of judging the tGP as a CW spiral is plotted against the CW dot contrast. The symbols demonstrate the averaged data points, whereas the curves show the model predictions described in the **Model** section in the **Discussion**. Lines with different colors refer to different CCW dot contrast levels. The vertical dashed lines indicate the contrast level of the anchor dot, which was set constant at −18dB contrast level in both conditions. The error bars are ±1 standard error of measurement.

Figure 2 shows results for the red or green tGPs with anchor dot contrast at −18 dB for three participants. Each row shows the data of one observer. The left panel contains data from the red condition, whereas the right panel, the green condition. The y-axis denotes probability judging a tGP as a CW spiral, while the x-axis shows the contrast of the CW dots. The smooth curves are the fits of our model (see the Discussion section). In each panel, different curves show data for a different CCW dot contrast. The dashed vertical line in each data plot indicates the contrast of the anchor dot. Thus, the data points where these dashed lines pass through are the results when the CW dot and anchor dot are at the same contrast level.

As the CW dot contrast increased, the probability for the participants to report the tGP as a CW spiral increased until a critical point. The curves then reached a plateau with few, if any, further increases in the chance of seeing the CW spiral. Such plateau probability decreased with the CCW dot contrast.

**Figure 3.**
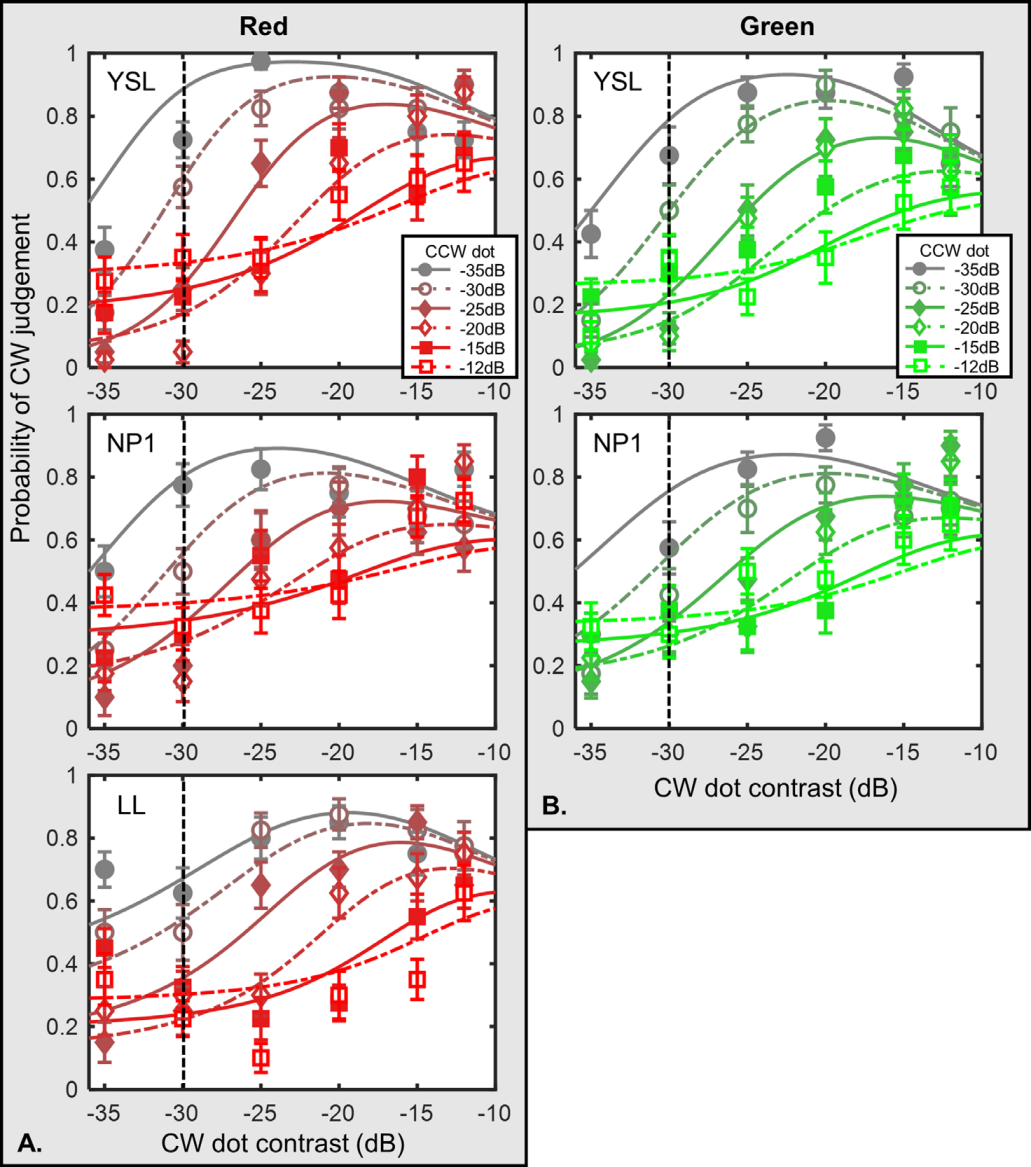
Results of red and green conditions with low-contrast anchor dots. Note: The figure shows the results of the red and green conditions with −30 dB anchor dot. (**A**) Data from three observers of the red condition. (**B**) Data from two observers in the green condition. Probability of judging the tGP as a CW spiral is plotted against the CW dot contrast. The symbols demonstrate the averaged data points, while the curves show the model predations described in the Model section in the Discussion. Lines with different colors refer to different CCW dot contrast levels. The vertical dashed lines indicate the contrast level of the anchor dot, which was set constant at −30 dB contrast level in both conditions. The error bars are ±1 standard error of measurement.

Reducing anchor dot contrast produced a very different picture. Figure 3 shows the results for the conditions with a −30 dB anchor dot contrast. The range of context dot contrast was from −35 to −12 dB. At low CW dot contrast, the probability of perceiving a CW spiral increased with CW contrast. This was similar to the results of the high contrast anchor condition. However, as the CW dot contrast further increased, the probability of seeing a CW spiral started to decrease, leading to an inverted-U shape curve. Notice that, in Figure 3, we only show the data from Yin-Shiuan Lin, NP1, and Lee Lin. Observer NP2 had difficulty seeing the anchor data due to the low contrast level, thus did not participate in the low-contrast anchor conditions. Observer Lee Lin only took part in the red condition.

### Blue and yellow colors

**Figure 4.**
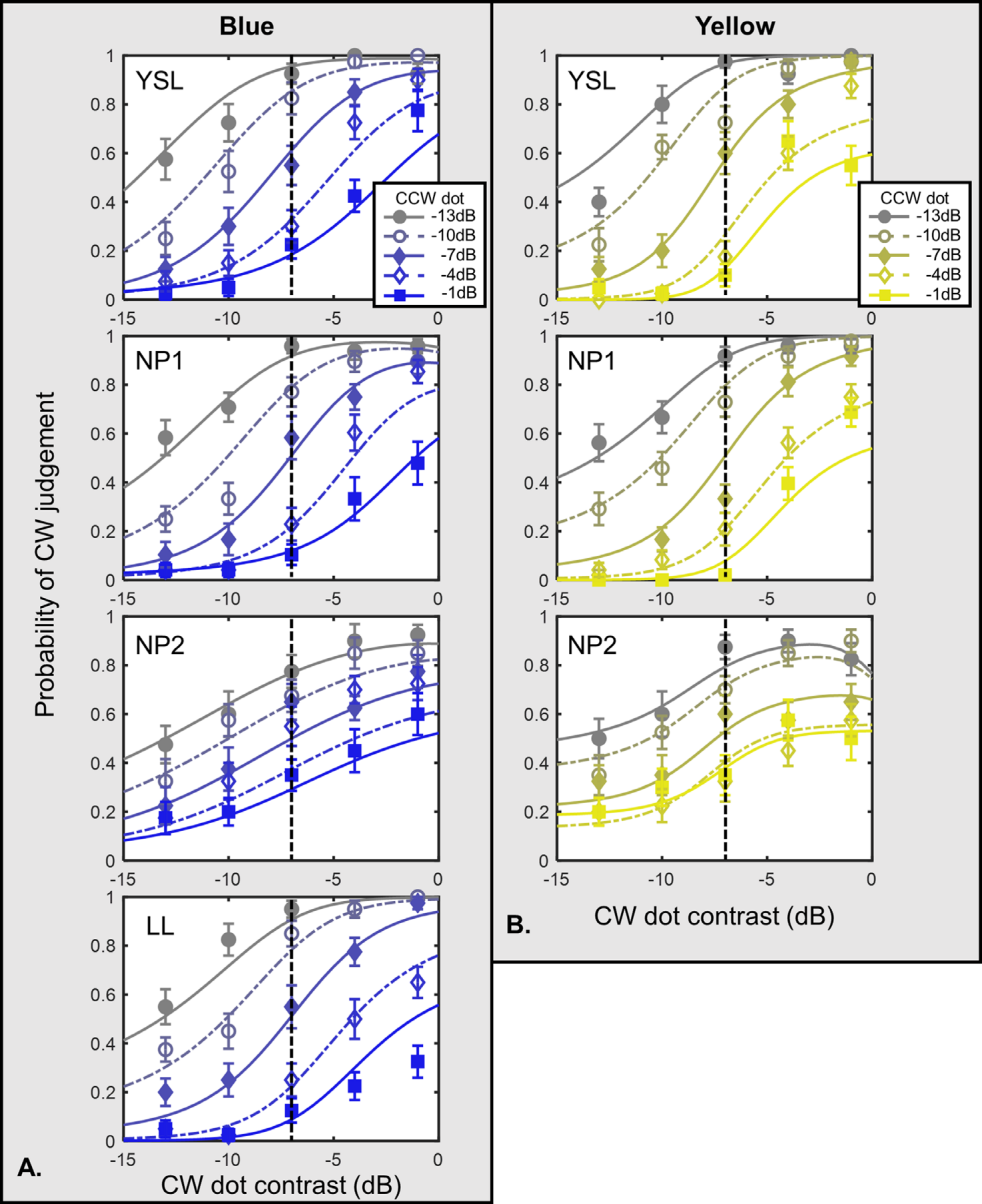
Results of blue and yellow conditions. Note: This figure represents the results of the blue and yellow conditions with −7 dB anchor dot. (**A**) Data from four observers of the blue condition. (**B**) Data from three observers of the yellow condition. Probability of judging the tGP as a CW spiral is plotted against the CW dot contrast. The symbols demonstrate the averaged data points, whereas the curves show the model predations described in the Model section in the Discussion. Lines with different colors refer to different CCW dot contrast levels. The vertical dashed lines indicate the contrast level of the anchor dot, which was set constant at −7 dB contrast level in both conditions. The error bars are ±1 standard error of measurement.

Figure 4 shows the result for the blue or yellow tGP conditions with anchor contrast −7 dB. The result was similar to the red or green tGP conditions at high anchor dot contrast. The probability of judging the CW increased with the contrast of the CW dot, and reached a plateau after a certain critical point. The plateau probability decreased with the CCW dot contrast.

## Discussion

In this study, we adopted the tGP paradigm of Lin et al. (2017) to study the role of color in grouping perception. That is, by grouping dots in a tripole in a different way, an observer may perceive different global forms. For example, a CW spiral is perceived as a result of the grouping of all CW dipoles. Thus, the final percept of the tripole Glass pattern is the result of the competition among these global forms. We manipulated the color contrast of the two context dots (CW and CCW dots) and observed how this manipulation affects such competition that determines the final global percept.

In the red or green conditions, for the same CCW contrast, the probability of seeing a CW spiral global pattern increased with the CW dot color contrast until a critical value. When the CW dot contrast further increased away from the anchor dot contrast, the probability for perceiving the CW decreased. Such an inverted-U shape, as discussed below, suggested a gain control process in play. The probability for seeing the CW spiral decreased with the CCW dot contrast, indicating that there was a greater chance of the CCW dot being grouped with the anchor dot. This suggests a competition between the CW and CCW spiral global grouping processes.

As for the blue or yellow conditions, similarly, the CW judging probability increased with the CW dot contrast and decreased with the CCW dot contrast, demonstrating inhibition between competing patterns. Due to the limited contrast range in the blue or yellow conditions, the contrast distance between the anchor dot and the context dots was not as large as in the red or green conditions. Thus, we could not observe the inverted-U shape when the CW dot contrast moved further away from the anchor dot contrast.

According to the energy model (Prazdny, 1984) of Glass pattern perception, an observer has a tendency to match dots with higher contrast together to form a dipole. In the context of our experiment, the energy model would predict that it would be easier for an observer to group the anchor dot with the context dot with higher contrast, resulting in a monotonic increase of CW judgment probability as the CW dot contrast increases. Therefore, this model cannot explain the inverted-U shape function when the anchor contrast was low in the red or green condition, where the CW judging probability actually began to decrease as CW dot contrast reached a critical point, between −25 and −20 dB.

Another alternative model for Glass pattern perception is the token model, or the similarity theory (Earle, 1999; Wilson et al., 2004), which suggests that the likelihood of two dots being grouped together increases when they are more similar to each other. As a result, the similarity theory would predict that regardless of the CCW dot contrast, the CW probability should always peak at the point where the CW dot and the anchor dot had the same contrast. However, in our data, the peaks shifted as the CCW dot contrast increased.

Neither the energy model nor similarity theory can provide a prediction that are inconsistent with the observed data. Lin et al. (2017) attempted to explain the global percept in tGPs with a contrast gain control model. Here, we extended this model to the chromatic stimuli. Despite the difference of spatial properties between the luminance and color vision mechanisms (Granger & Heurtley, 1973; Kelly, 1975; Mullen, 1985; van der Horst & Bouman, 1969; Webster et al., 1990), different roles played by luminance and color in visual illusions and in the grouping perception (Li & Guo, 1995; McIlhagga & Mullen, 1996; Mullen et al., 2000; Mullen & Beaudot, 2002), we expect our model can explain the global percept on equiluminance tGPs.

### Model

**Figure 5.**
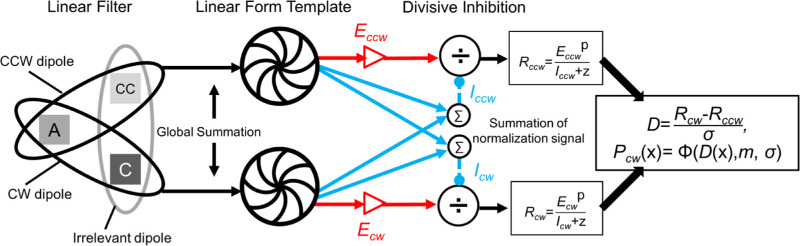
Contrast gain control model. Note: This figure illustrates the model used in the current study, modified from Figure 5 in Lin et al. (2017). See text in the Introduction and Discussion sections for a detailed description.

We fitted a divisive inhibition model, which describes a contrast gain control mechanism, to our data. In our model, we first calculated the responses toward all possible dipoles in one local tripole, which were a CCW dipole, a CW dipole, and an irrelevant dipole, as shown in the schematic diagram in Figure 5. The neural activation of one dipole is affected by the area the two dots cast on the receptive field targeting the dipole. Thus, the activation toward one dipole is determined by both the spatial factor (size of the dots) and the contrast factor (in our case, color contrast).

Because the spatial factor is fixed in the current study (dot size was the same for all conditions), whereas the contrast factor changed with different dot contrasts, a spatial factor independent of contrast could be seen as a sensitivity of neurons towards contrast change of the dipole. That is, the activation we had measured was the result of dipole contrast multiplying contrast sensitivity. We then assumed a linear global template that summed up the excitation of all local dipoles that conform to the orientation of a global form. Therefore, in our model, the excitatory activation of one such global template is defined as the sensitivity times the color contrast:

(1) $$E_{j}=Se_{j}\times Cd_{j},$$

and the inhibitory activation is defined as

(2) $$I_{j}=\sum_{k=1}^{n}{\left(Si_{k}\times Cd_{k}\right)}^{q}.$$

*Se_j_* represents the excitatory sensitivity of the *j*-th global template (in our case, the CCW spiral, CW spiral, or irrelevant pattern). *Cdj* is the contrast of the dipoles that conform to the orientation of the *j*-th global form, which was calculated by summing up the contrasts of both dots in one dipole. In Equation 2, *q* is the power parameter, and *Si_k_* is the inhibitory sensitivity of the *k*-th global form among the three aforementioned global templates.

Taking the response to the CW spiral as an example, in Equation 3, *R_cw_* is composed of an excitatory component in the numerator (*E_cw_*), and an inhibitory component (*I_cw_*), as well as a normalizing parameter (*z*) in the denominator. That is:

(3) $$\begin{array}{c} R_{cw}=\frac{{E_{cw}}^{p}}{I_{cw}+z}.\; \end{array}$$

The *E_cw_* is determined by the contrast of the CW dipole, *C_cw_* (here, the sum of contrast values from both dots within the CW dipole), and the sensitivity toward the CW dipole, *Se_cw_*, as in

(4) $$\begin{array}{c} E_{cw}=Se_{cw}\times Cd_{cw}.\; \end{array}$$

We assumed that observers did not have bias toward either the CW or CCW spirals, thus we later used one parameter *Se* for both CW and CCW sensitivity. *I_cw_* is defined as the sum of *C_cw_* and the contrasts of the other dipoles *C_ccw_* and *C_irre_* after timing their sensitivity coefficients, *Si_t_*, *Si_1_*, and *Si_2_*, respectively, that is,

(5) $$\begin{array}{c} \;\;\;\;\;\;\;\;\;\;\;\;\;\;\;I_{cw}=\;{\left(Si_{t}\times Cd_{cw}\right)}^{q}+{\left(Si_{1}\times Cd_{ccw}\right)}^{q}+{\left(Si_{2}\times Cd_{irre}\right)}^{q}.\; \end{array}$$

*Si_t_* is the inhibition sensitivity from the corresponding global form, whereas *Si_1_* and *Si_2_* are those from the rest of the two global form templates (in this example, the CCW spiral and irrelevant pattern). *Cd_cw_*, *Cd_ccw_*, and *Cd_irre_* correspond to the contrast of the CW dipole, CCW dipole, and the irrelevant dipole, respectively. We later discovered that removing the irrelevant global form template from the equation did not affect the model performance, thus we set the parameter *Si_2_* as 0.

The final decision was determined by the difference between the response to the CW and that to the CCW responses, or

(6) $$\begin{array}{c} D_{cw}=R_{CW}-R_{CCW}\; \end{array}$$

The CW judging probability (*Pcw*) was determined by a Gaussian cumulative distribution function (cdf), *Φ*, or

(7) $$\begin{array}{c} Pcw\left(x\right)=\Phi \left(D_{cw}\left(x\right),m,\;\sigma \right),\; \end{array}$$

where *m* is the location parameter (“mean”) and *σ* is the scale parameter (“standard deviation”) of the Gaussian function. Parameter *m* represents observer's response bias: A negative *m* suggests the observer was more inclined to make a CW judgement and a positive *m*, a CCW judgement. The *σ* was set to one throughout the model fitting.

We used the Powell's algorithm (Press, Teukolsky, Vetterling, & Flannery, 1988) to optimize the parameter values that minimized the sum of squared error, or the sum of the squared deviations between the model prediction and the data. Tables 1 and 2 show the fitting parameters and goodness of fit (*R^2^*) for all observers in red/green and blue/yellow conditions. Except for *Si_t_*, *Si_1_*, *q*, *z*, and *m*, all other parameters fixed for *R^2^* did not differ empirically. In Lin et al. (2017), we did not observe a response bias towards CW or CCW judgment in the isochromatic data, therefore, the *m* parameter was set to be fixed at 0. In the current study, we noticed that letting *m* to be a free parameter significantly improved the goodness-of-fit. Therefore, we chose to fit the current equiluminance data set with the model with five free parameters instead of with four in Lin et al. (2017). The value of parameter *p* and *σ* were set to 1. Doing so also did not affect the goodness-of-fit. Overall, our model explained 77.50% to 97.73% of the variance in the data, with rooted mean square errors (RMSE) ranging from 0.0417 to 0.1012 and mean standard error (MSE) from 0.0386 to 0.0711, across all observations and conditions.

**Table 1.** List of fitting parameters and R^2^ for all participants in the red and green conditions.

	Observers		
	YSL	NP1	NP2
	
**Parameters**	**Red [−18 dB anchor]**		
P	1.00^a^	1.00^a^	1.00^a^
Q	5.07	4.55	6.25
Z	1.48	0.00	14.03
Se_t_	100.00^a^	100.00^a^	100.00^a^
Si_t_	0.00	3.34	3.57
Si_1_	7.39	8.56	7.47
Si_2_	0.00^a^	0.00^a^	0.00^a^
M	**−**0.08	**−**0.24	**−**0.15
*σ*	1.00 ^a^	1.00 ^a^	1.00 ^a^
*R^2^* (%)	96.66	97.73	96.72
	**Green** **(−18 dB anchor)**		
P	1.00^a^	1.00^a^	1.00^a^
Q	6.43	5.08	3.71
Z	3.89	2.90	0.02
Se_t_	100.00^a^	100.00^a^	100.00^a^
Si_t_	0.02	0.02	5.96
Si_1_	6.25	7.78	10.50
Si_2_	0.00^a^	0.00^a^	0.00^a^
M	**−**0.20	**−**0.12	**−**0.21
*σ*	1.00 ^a^	1.00 ^a^	1.00 ^a^
*R^2^* (%)	96.60	97.60	89.83

	YSL	NP1	LL

	**Red** **(−30 dB anchor)**		
P	1.00^a^	1.00^a^	1.00^a^
Q	3.05	2.55	2.56
Z	1.39	0.00	3.52
Se_t_	100.00^a^	100.00^a^	100.00^a^
Si_t_	11.62	16.63	14.11
Si_1_	23.84	15.76	18.31
Si_2_	0.00^a^	0.00^a^	0.00^a^
M	**−**0.25	**−**0.15	**−**0.11
*σ*	1.00 ^a^	1.00 ^a^	1.00 ^a^
*R^2^* (%)	89.55	77.50	84.51
	**Green** **(−30 dB anchor)**		
P	1.00^a^	1.00^a^	
Q	2.66	2.21	
Z	0.49	0.19	
Se_t_	100.00^a^	100.00^a^	
Si_t_	14.33	19.84	
Si_1_	28.90	24.39	
Si_2_	0.00^a^	0.00^a^	
M	0.00	**−**0.13	
*σ*	1.00 ^a^	1.00 ^a^	
*R^2^* (%)	85.97	79.88	

*Note.* ^a^Fixed parameters.

**Table 2.** List of fitting parameters and R^2^ for all participants in blue and yellow conditions.

Observers				
	YSL	NP1	NP2	LL
	
**Parameters**	**Blue** **(−7 dB anchor)**			
p	1.00^a^	1.00^a^	1.00^a^	1.00^a^
q	3.07	4.06	3.87	5.37
z	5.87	13.48	25.77	30.74
Se_t_	100.00^a^	100.00^a^	100.00^a^	100.00^a^
Si_t_	2.24	1.78	1.78	1.02
Si_1_	2.40	2.12	4.05	2.31
Si_2_	0.00^a^	0.00^a^	0.00^a^	0.00^a^
m	**−**0.28	**−**0.03	0.00	**−**0.03
*σ*	1.00 ^a^	1.00 ^a^	1.00 ^a^	1.00 ^a^
*R^2^* (%)	96.59	97.08	87.51	96.78

	YSL	NP1	NP2	

	**Yellow** **(−7 dB anchor)**			
p	1.00^a^	1.00	1.00^a^	
q	7.22	6.651	11.62	
z	29.79	34.73	83.72	
Se_t_	100.00^a^	100.00^a^	100.00^a^	
Si_t_	0.47	0.13	1.05	
Si_1_	2.00	2.02	1.77	
Si_2_	0.00^a^	0.00^a^	0.00^a^	
m	**−**0.19	**−**0.02	**−**0.07	
*σ*	1.00 ^a^	1.00 ^a^	1.00 ^a^	
*R^2^* (%)	94.78	96.61	94.32	

*Note.* ^a^Fixed parameters.

Such fitting results showed that this variant of the contrast gain control model can successfully explain the data variance. The self-inhibition term in Equation 5 (*Si_t_*) is crucial as it shows that the response of, for example, CW pattern detectors can decrease with the contrast of the CW dot. This property allows the inverted-U shape function in our data that cannot be explained by the energy model (Prazdny, 1984). Additionally, the inhibition term from the other global form (*Si*_1_) in Equation 5 allows the location and shape of the inverted-U shape function to be influenced by the contrast of the other context dot in the tripole. It is necessary to capture the peak shifts observed in the data. Such a feature cannot be captured by the token model or similarity theory (Earle, 1999; Wilson et al., 2004). Therefore, compared to the competing models, our divisive inhibition model offers better predict and explains all the major features of the data.

The inverted-U curves and the shifting peak can be explained by the self-inhibitory and other inhibitory components in our model, respectively. First, as the contrast of dipoles in one global form increased, the strength of self-inhibition increased exponentially. The resulting larger contribution of inhibitory component in the denominator in Equation 3 led to a weakened response toward this global template and reduced the probability of judging the tGP as the current global form.

Second, the value of the other inhibitions increased with the contrast value in other competing global templates. Increase of competing contrast of the competing global templates led to a weakened response toward the target global form. This can explain why the peaking CW judging probability shifted to the right as the contrast of the CCW dot increased in low anchor contrast conditions (see Figure 3).

## Comparison of nominal and subjective equiluminance

Our stimuli, as shown in the Methods section, was nominal equiluminance as the chromaticity was computed (Derrington, Krauskopf, & Lennie, 1984; Krauskopf, Williams, & Heeley, 1982; MacLeod & Boynton, 1979) based on the cone fundamental (CIE, 2006). Due to the individual difference, there might be a discrepancy between the nominal equiluminance of our stimuli and the subjective equiluminance setting of individual observers. This discrepancy may introduce a residual luminance contrast in our stimuli. Some may argue that our results may be due to such residual luminance contrast. In particular, our low anchor contrast result showed a similar functional to the luminance contrast in Lin et al. (2017). However, notice that such argument ignored the well-established fact that human observers are more sensitive to L-M contrast than luminance one (Chaparro, Stromeyer, Huang, Kronauer, & Eskew, 1993; King-Smith & Carden, 1976; Mullen, 1985; Mullen, Thompson, & Hess, 2010; Párraga, Troscianko, & Tolhurst, 2002; Watson, Barlow, & Robson, 1983). It is unlikely that an observer should ignore a strong signal from the L-M contrast but make a response based on a weak signal from luminance contrast. Nevertheless, we empirically estimated the effect of the possible residual luminance contrast on our result. We first estimated the subjective equiluminance point of two observers (authors Y.S.L. and L.L.) with heterochromatic flicker photometry (see Appendix B for the experimental method and Table B1 for the results). The estimated residual luminance contrast for the three observers were either below or near luminance contrast threshold even at the highest contrast we used. Thus, the residual luminance contrast resulted from the discrepancy between the subjective and the nominal equiluminance is negligible.

**Figure 6.**
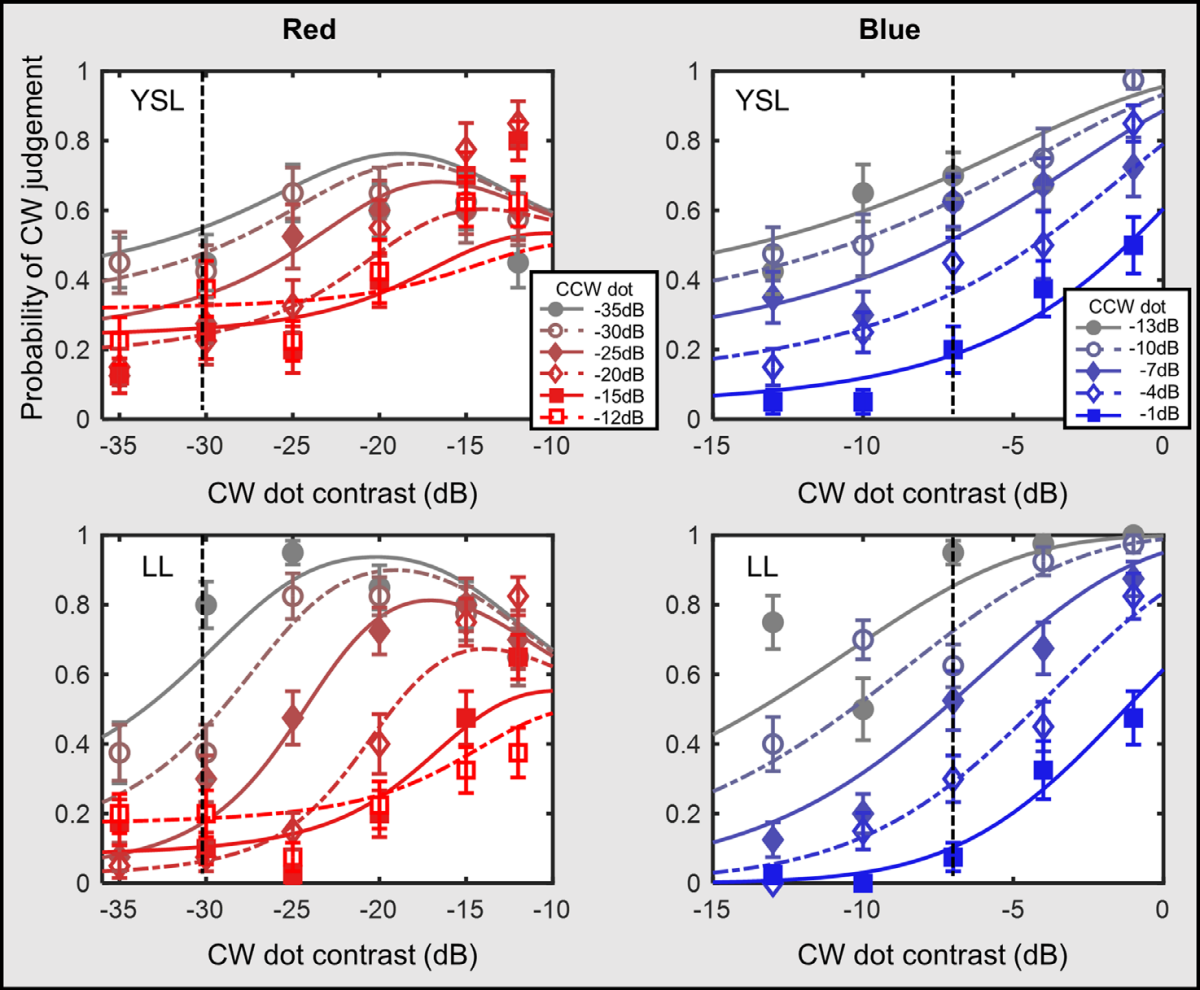
Results of participants Yin-Shiuan Lin and Lee Lin with subjective equiluminance setting. Note: This figure shows the results of the red (left panel) and blue (right panel) conditions in the subjective (right column) equiluminance of participants Yin-Shiuan Lin and Lee Lin. The anchor dot contrast, indicated by the dashed-vertical lines, was of −30 dB in the red condition and −7 dB in the blue condition. The symbols denote the averaged data points whereas the curves are the predations of the model described in the Model section in the Discussion section. Lines with different colors refer to different CCW dot contrast levels. The error bars are ±1 standard error of measurement.

Two observers (YSL and LL) repeated the low anchor contrast conditions in both red and blue colors with their own subjective equiluminance setting. There was no systematic difference between subjective (Figure 6) and nominal (see Figure 3, red condition, and Figure 4, blue condition) equiluminance results. Statistically, there was also no significant difference between the nominal and the subject equiluminance datasets (YSL: χ^2^= 36.38 and 21.34, df = 35 and 24, *p* = 0.40 and 0.62; LL: χ^2^= 38.39 and 19.84, df = 35 and 24, *p* = 0.32 and 0.71 for red and blue, respectively). Thus, the residual luminance contrast played no role in our result.

## Comparison between equiluminance and isochromatic data

**Figure 7.**
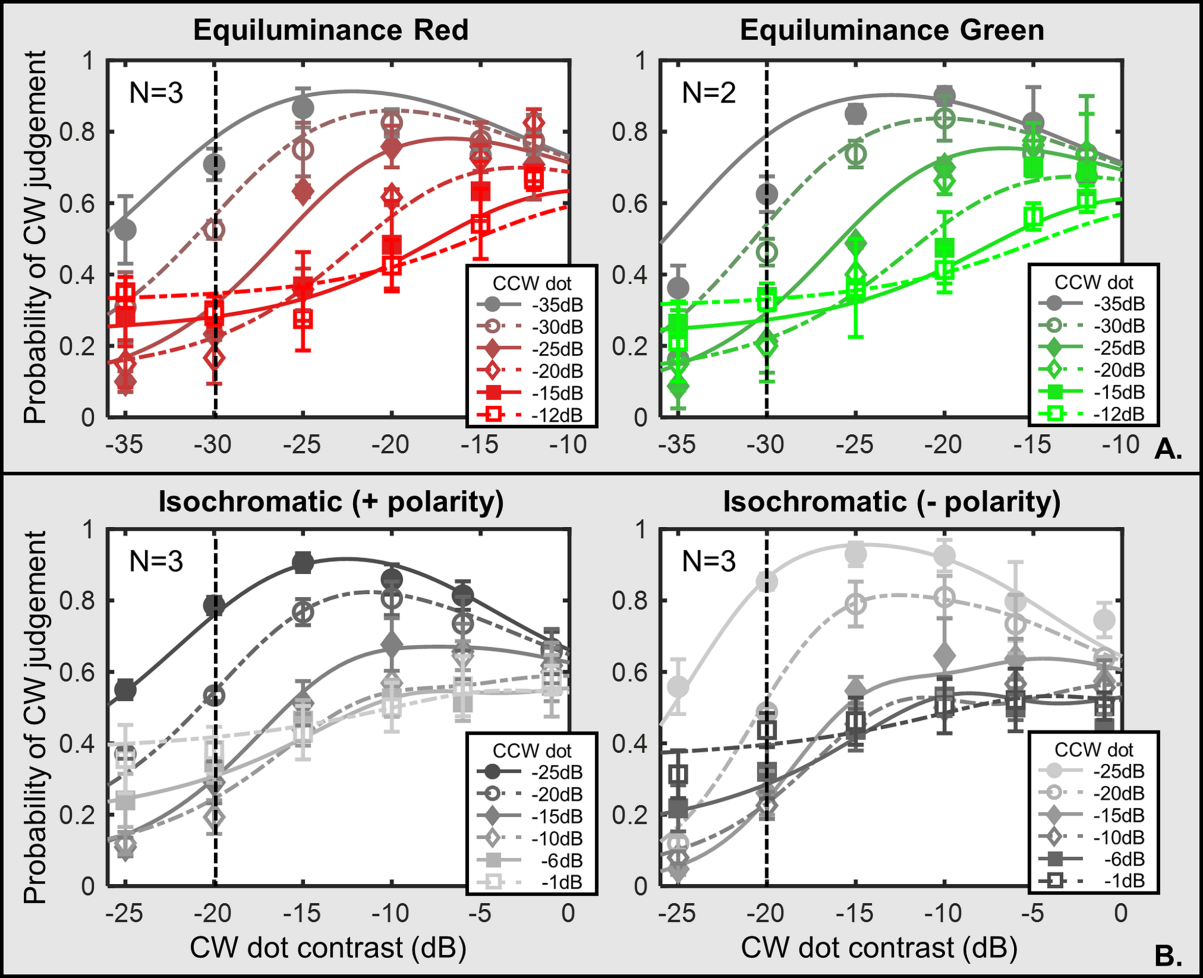
Comparison between chromatic and luminance data. Note: (**A**) Average data of the observers (three in the red condition and two in the green condition) in equiluminance conditions. The anchor dot was at −30 dB. (**B**) Averaged data of three observers from Lin et al. (2017) in isochromatic. Anchor dot contrast was at −20 dB. The error bars are ±1 standard error of measurement.

Next, we investigated whether color contrast contributed differently from luminance contrast in the Glass pattern percept. We compared averaged data of this study, and those reported in Lin et al. (2017). For the luminance condition, we chose a partial data set so that both conditions had similar contrast range (−35 to −10 dB for chromatic and −25 to −1 dB for luminance condition). Model fitting results are shown in Figure 7, and the fitting parameters are demonstrated in Table 3. Model predictions accounted for an 89.58% to 97.85% variance in the data, with RMSEs ranging from 0.0296 to 0.0752 and MSE between 0.0512 and 0.0587.

**Table 3.** List of fitting parameters and R^2^ in equiluminance and isochromatic conditions.

	Condition	
	Equiluminance	
Parameters	Red	Green
p	1.00^a^	1.00^a^
q	2.39	2.29
z	0.83	0.15
Se_t_	100.00^a^	100.00^a^
Si_t_	16.80	18.10
Si_1_	20.98	21.54
**Si_t_/Si_1_**	**0.80**	**0.84**
Si_2_	0.00^a^	0.00^a^
m	**−**0.15	**−**0.10
*σ*	1.00^a^	1.00^a^
*R^2^* (%)	94.09	89.58

	**Isochromatic**	
	
	**Positive polarity**	**Negative polarity**
p	1.00^a^	1.00^a^
q	3.29	3.41
z	5.06	2.12
Se_t_	100.00^a^	100.00^a^
Si_t_	4.88	4.59
Si_1_	3.89	3.67
**Si_t_/Si_1_**	**1.25**	**1.25**
Si_2_	0.00^a^	0.00^a^
m	**−**0.11	**−**0.06
*σ*	1.00^a^	1.00^a^
*R^2^* (%)	97.77	97.85

*Note.* ^a^Fixed parameters.

The inverted-U trend was less noticeable in the chromatic than in the luminance condition. The inhibitory components in our divisive inhibition model can account for this difference. The ratio between *Si_t_* and *Si_1_* (*Si* ratio) indicates the strength of self-inhibition relative to other inhibitions: the larger the ratio, the stronger the self-inhibition. The *Si* ratios in equiluminance condition were smaller than in isochromatic condition (see bold numbers in Table 3).

## Conclusion

To the best of our knowledge, we are the first study to investigate the role of color contrast in Glass pattern perception when multiple grouping possibilities are presented simultaneously. Two significant findings were discovered in our results. First, similar to what we found in isochromatic experiments (Lin et al., 2017), the probability of one of the context dots grouped with the anchor dot increased with the color contrast of that context dot, then decreased after a critical level, resulting in an inverted-U shape.

Second, the grouping probability decreased as the contrast of the other context dot increased (in low anchor condition). Based on both equiluminance and isochromatic results, we concluded that the contrast gain control mechanism was presented in both situations. However, the inhibition components differed between stimuli under luminance and chromaticity manipulation. Contribution of self-inhibition was found to be weaker than inhibition from others when we manipulated chromaticity (see Figure 7, Table 3). The difference we found fit well with other research findings supporting that luminance contrast and chromatic contrast play different roles in spatial vision.

## Appendix A: Threshold measurement

To determine the appropriate contrast range for our stimuli, we measured the contrast thresholds to our stimuli. The procedure for threshold measurement was similar to the main experiment (see Methods section) except that only one of the context dots was presented with the anchor dot and that the two dots were always with the same contrast level in each trial. The contrast ranged from −40 dB to −28 dB for red and green, −19 dB to −7 dB for blue and yellow, and −32 dB to −20 dB for luminance contrast (+ polarity). The probability of correctly judging the stimulus as CW or CCW spiral was fit with a cumulative normal psychometric function. The threshold was defined as the contrast level that produced 75% correct response of the observer. The estimated thresholds in dB unit for observer Yin-Shiuan Lin, Lee Lin, and NP1 are listed in the Table A1.

**Table A1.** The estimated thresholds in dB unit for observer yin-shiuan lin, Lee Lin, and NP1.

	Red	Green	Blue	Yellow	Luminance
YSL	**−**33.90	**−**33.09	**−**13.98	**−**14.77	**−**26.91
LL	**−**30.91	**−**32.18	**−**13.75	**−**14.06	**−**26.52
NP1	**−**31.90	**−**31.25	**−**14.68	**−**14.24	**−**24.45

LL, Lee Lin, YSL, Yin-Shiuan Lin.

## Appendix B: Experimental procedure for measuring subjective equiluminance points

We conducted a variation of the heterochromatic flicker photometry test to measure the subjective equiluminance point of the colors used in the main experiment for observer Yin-Shiuan Lin and Lee Lin. In each trial, a sinusoidal grating oscillated between two opponent colors (the color contrast was −18 dB for the red/green grating and −2 dB for the blue/yellow grating) flickered in counter phase at 15 Hz for 267 ms (16 frames). Participant was to press the corresponding buttons on the keyboard to adjust the elevation of the stimuli in the color space (see the Methods section), that is, the luminance difference between the two colors of the sinusoidal grating until the perceived flickering was minimized. Each color pair was repeated for ten times. Table B1 shows (1) the estimated elevation at the minimum flicker for red/green (column 2) and blue/yellow directions (column 4), (2) the corresponding luminance contrast difference between the subjective and the nominal equiluminance at the highest color contrast (−12 dB for red/green and −1 dB for blue/yellow) used in the main experiment, and (3) the luminance detection threshold (see Appendix A for the experimental method).

**Table B1.** The results of HFP test of participants yin-shiuan Lin and lee Lin.

	Red		Blue		
	Estimated elevation	Residual luminance contrast	Estimated elevation	Residual luminance contrast	Luminance threshold
YSL	**−**11.50 degrees	**−**26.00 dB	1.01 degrees	**−**40.69 dB	**−**26.91 dB
LL	6.19 degrees	**−**31.34 dB	0.59 degrees	**−**36.09 dB	**−**26.52 dB
NP1	**−**4.90 degrees	**−**33.33 dB	**−**0.35 degrees	**−**45.26 dB	**−**24.45 dB

LL, Lee Lin, YSL, Yin-Shiuan Lin.
